# Body Roundness Index and A Body Shape Index as First-Trimester Risk-Stratification Markers for Gestational Diabetes in Overweight Pregnant Women Without Established Major Risk Factors: A Prospective Single-Center Cohort Study

**DOI:** 10.3390/diagnostics16152384

**Published:** 2026-07-29

**Authors:** Ömer Osman Eroğlu, Cansın Eroğlu, Sait Erbey, Murat Polat, Bilge Erbey, Mehmet Alican Sapmaz, Çağanay Soysal

**Affiliations:** 1 Department of Obstetrics and Gynecology, Ankara Etlik City Hospital, Ankara 06170, Türkiyesaiterbey@gmail.com (S.E.); dr.alicansapmaz@hotmail.com (M.A.S.);; 2 Independent Researcher, Ankara 06800, Türkiye; blgegoktas@gmail.com

**Keywords:** gestational diabetes mellitus, Body Roundness Index, A Body Shape Index, first-trimester screening, risk stratification, anthropometric biomarkers, overweight pregnancy, diagnostic accuracy

## Abstract

**Background/Objectives:** Universal screening for gestational diabetes mellitus (GDM) is performed at 24–28 gestational weeks, but recommendations for earlier risk stratification differ across guidelines. Overweight pregnant women (body mass index [BMI] 25.0–29.9 kg/m^2^) without established major GDM risk factors constitute a clinical gray zone for which current guidelines provide no explicit directive. We evaluated the first-trimester diagnostic performance of two composite anthropometric indices—the Body Roundness Index (BRI) and A Body Shape Index (ABSI)—for subsequent IADPSG-defined GDM within this specific clinical niche. **Methods:** This prospective single-center cohort study enrolled 198 consecutive overweight pregnant women without major GDM risk factors at 11 + 0 to 13 + 6 gestational weeks; 186 participants completed the 75 g OGTT at 24–28 weeks and were analyzed. Anthropometric measurements were obtained by trained nurses blinded to outcome. Glucose was assayed by hexokinase enzymatic reference method (intra-assay CV ≤ 1.5%). Discrimination, calibration, reclassification (NRI, IDI), decision curve analysis, and three independent internal validation procedures were performed. **Results:** GDM prevalence was 18.28% (34/186). BRI achieved an AUC of 0.905 (95% CI 0.835–0.974) and ABSI an AUC of 0.889 (95% CI 0.815–0.963), both significantly greater than BMI (AUC 0.705; DeLong *p* < 0.001). The optimal BRI cutoff of 4.45—interpreted as a cohort-specific analytic anchor rather than a transferable clinical threshold—yielded 85.3% sensitivity and 90.8% specificity. Adding BRI to BMI improved discrimination by 45.6% (IDI). Bootstrap optimism remained below 0.01 across all models. **Conclusions:** Following internal validation through bootstrap optimism correction and cross-validation, first-trimester BRI and ABSI provided strong supplementary risk stratification for GDM in overweight pregnant women without major risk factors. External validation in independent populations is required before clinical implementation.

## 1. Introduction

Gestational diabetes mellitus (GDM) is defined as glucose intolerance with onset or first recognition during pregnancy and represents one of the most common medical complications of pregnancy worldwide. Affected women face elevated risks of pregnancy-related complications including pre-eclampsia, operative delivery, shoulder dystocia, and birth trauma, while their offspring are at increased risk of macrosomia, neonatal hypoglycemia, neonatal intensive care unit admission, and longer-term metabolic dysregulation. The landmark Hyperglycemia and Adverse Pregnancy Outcome (HAPO) study established a continuous, graded relationship between maternal glucose levels and adverse perinatal outcomes even below previous diagnostic thresholds, fundamentally reshaping the conceptual framework of pregnancy-associated glucose intolerance [1]. Building on HAPO findings, the International Association of Diabetes and Pregnancy Study Groups (IADPSG) introduced harmonized diagnostic criteria for the one-step 75 g oral glucose tolerance test, which have since been adopted by major obstetric and diabetes societies internationally [2].

The global prevalence of GDM varies widely depending on diagnostic criteria, population characteristics, and screening strategies, with reported rates ranging from approximately 5% to more than 20% of pregnancies. In Türkiye, GDM prevalence has been reported in the range of 14–17% under contemporary diagnostic frameworks [3]. Beyond its immediate perinatal impact, GDM carries enduring clinical significance: affected women have a substantially elevated lifetime risk of progression to type 2 diabetes mellitus, cardiovascular disease, and metabolic syndrome, while their children face increased susceptibility to childhood obesity and impaired glucose metabolism. These short- and long-term consequences underscore the importance of timely identification and risk-stratified management.

Current clinical practice for GDM screening rests on a universal one-step or two-step approach performed at 24–28 gestational weeks, supported by both the IADPSG and major obstetric societies [2,4]. The role of early (first-trimester) screening, however, remains considerably less harmonized across guidelines. The American Diabetes Association recommends early testing for women at high risk of undetected pre-existing diabetes or those carrying at least one established risk factor for GDM, including obesity, prior GDM, first-degree family history of diabetes, prior macrosomic infant, polycystic ovary syndrome, or evidence of insulin resistance [5]. In contrast, the American College of Obstetricians and Gynecologists adopts a more conservative position and does not endorse early universal screening based on overweight status alone in the absence of additional risk factors [4].

Recent prospective evidence has begun to clarify the potential benefit of early screening in selected populations. The Treatment of Booking Gestational Diabetes Mellitus (TOBOGM) randomized trial demonstrated that immediate treatment of early-pregnancy hyperglycemia among women with at least one GDM risk factor reduced a composite adverse neonatal outcome compared with deferred treatment, providing the strongest contemporary evidence that early identification can translate into measurable clinical benefit [6]. Nevertheless, both the TOBOGM trial and the ADA recommendations are anchored on the presence of at least one established major risk factor as the eligibility threshold for early screening.

This guideline architecture leaves an important clinical gray zone—defined here as a clinical situation in which existing guidelines offer no explicit or consistent screening directive: pregnant women who are overweight (BMI 25.0–29.9 kg/m^2^) but who do not carry any of the established major risk factors for GDM. In this population, current ACOG guidance does not recommend early screening, ADA criteria do not formally apply, and the TOBOGM evidence base does not directly extend. Yet this subgroup constitutes a substantial proportion of contemporary obstetric populations and exhibits intermediate metabolic risk that may be inadequately captured by BMI alone. Although overweight status itself confers elevated GDM risk and is recognized as a screening consideration in several guidelines, the distinct clinical question of how to further stratify risk within the overweight population—particularly in the absence of additional established risk factors—remains unaddressed; the present study was designed to address this question of within-overweight risk stratification. Practical and inexpensive first-trimester risk stratification tools designed specifically for this gray zone could therefore complement existing guidelines without altering universal screening recommendations.

Anthropometric assessment in early pregnancy offers a practical entry point for first-trimester risk stratification. The necessary measurements—height, weight, and waist circumference—are inexpensive, non-invasive, and already integrated into routine antenatal care. The first trimester is particularly well suited for this purpose, as the gravid uterus remains confined within the pelvic cavity and does not yet meaningfully distort abdominal anthropometric measurements. While BMI is the most widely used index, it captures only the relationship between weight and height and does not distinguish between subcutaneous and visceral adipose tissue or reflect body fat distribution—a limitation of particular relevance to GDM, in which visceral adiposity and centrally distributed fat are implicated in insulin resistance pathophysiology [7].

Two composite anthropometric indices have been developed to address these limitations. The Body Roundness Index (BRI), proposed by Thomas et al. [8], integrates waist circumference and height into a geometric ellipse-based formulation designed to capture body roundness and, by extension, central adiposity. A Body Shape Index (ABSI), proposed by Krakauer and Krakauer [9], was constructed specifically to be mathematically independent of BMI, isolating waist circumference-based shape information from overall body size. This property is clinically relevant precisely in populations where BMI is a poor discriminator: because ABSI captures the component of central adiposity that BMI cannot, it can differentiate metabolic risk between individuals whose BMI values are similar—as is the case, by definition, within a narrowly defined overweight cohort—thereby offering information of potential value for GDM risk stratification that BMI alone would miss. Both indices have been shown to outperform BMI for the prediction of metabolic and cardiovascular outcomes in non-pregnant populations [10]. Beyond the general population, both indices have attracted growing attention in obstetrics, where their predictive value extends beyond glucose metabolism to a range of adverse pregnancy conditions. Elevated first-trimester ABSI and BRI have been associated with unexpected recurrent pregnancy loss, with reported discriminatory cutoff values of 0.079 and 4.37, respectively [11]. Higher BRI has likewise been prospectively linked to incident hypertension in the general adult population [12], and, in pregnancy specifically, early central-adiposity indices—including waist-to-height ratio and BRI—have been associated with both gestational diabetes and hypertensive disorders of pregnancy, consistent with a shared adiposity-driven pathophysiology that BMI alone may not fully capture [13]. ABSI, in particular, is not intended to predict any single disease in isolation but rather to provide a body-size-independent measure of central shape that complements other anthropometric and clinical parameters. Collectively, these observations support the broader rationale for evaluating shape-based composite indices, rather than BMI alone, when seeking to refine first-trimester risk stratification in pregnancy.

In the obstetric setting, several studies have examined first-trimester BRI and ABSI for GDM prediction. A Turkish midgestation cohort reported an AUC of approximately 0.74 for BRI [14], while subsequent reports from diverse populations have described AUC values ranging broadly between 0.65 and 0.85 [15,16,17,18,19,20,21,22,23,24]. Most recently, Pape et al. [25], in a conference abstract presented at the American Diabetes Association Scientific Sessions, examined first-trimester BRI in a large multicenter U.S. cohort of nulliparous pregnant women (NuMoM2b, *n* = 9675) and reported an overall AUC of 0.69 for GDM prediction, with only a modest advantage over BMI (AUC 0.66); in the overweight subgroup specifically (*n* = 2410), the adjusted odds ratio for BRI ≥ 3.65 was 1.76 (95% CI 1.08–2.87). The authors concluded that BRI did not provide a substantive clinical advantage over BMI in their unselected population, an interpretation that is important to acknowledge when situating composite anthropometric indices within the broader screening landscape.

Two observations follow from this body of evidence. First, the reported discriminative performance of BRI for GDM varies considerably across populations, sampling frames, and trimester of measurement—reflecting the influence of cohort composition (parity, ethnicity, baseline risk distribution) and methodological heterogeneity. Second, no published study to date has specifically evaluated BRI and ABSI in the clinically defined gray zone described above: overweight pregnant women without established major risk factors for GDM. Evaluating composite anthropometric indices in this targeted population, rather than in unselected obstetric cohorts, may provide complementary information about their potential utility as a supplementary risk-stratification tool where current guidelines provide no explicit directive.

The central research question of this study was therefore explicit and singular: among overweight pregnant women who lack established major GDM risk factors—a group for whom current guidelines provide no clear early-screening directive—can first-trimester composite anthropometric indices (BRI and ABSI) discriminate those who will subsequently develop GDM more effectively than BMI alone? Against this background, we designed a prospective single-center cohort study to evaluate the first-trimester diagnostic performance of BRI and ABSI for subsequent GDM specifically within the clinically defined gray zone described above—pregnant women with BMI 25.0–29.9 kg/m^2^ and no established major risk factors for GDM. Our a priori hypothesis was that, in this targeted subgroup, BRI and ABSI would provide stronger discrimination for GDM than BMI alone, by capturing information related to central adiposity and body shape that is not reflected by BMI. The primary outcome was the diagnostic performance (AUC, sensitivity, specificity, and likelihood ratios) of each anthropometric index for IADPSG-defined GDM ascertained at 24–28 gestational weeks. Secondary objectives included quantifying reclassification improvement over a BMI-only model, evaluating clinical utility through decision curve analysis, and assessing the internal robustness of model performance through complementary internal validation procedures. The framing throughout this study positions BRI and ABSI as potential supplementary first-trimester risk-stratification tools for a specific guideline gray zone, complementary to—not a replacement for—universal second-trimester GDM screening.

## 2. Materials and Methods

### 2.1. Study Design and Ethics

This was a single-center, prospective, observational cohort study conducted at the Department of Obstetrics and Gynecology, Ankara Etlik City Hospital, Ankara, Türkiye. The study was approved by the Clinical Research Ethics Committee of Ankara Etlik City Hospital (Approval No: AEŞH-EK-2026-035; Approval Date: 4 February 2026) and was conducted in accordance with the principles of the Declaration of Helsinki. Given the prospective study design, written informed consent was obtained from all participants prior to enrollment, following a detailed explanation of the study aim, procedures, the voluntary nature of participation, and measures ensuring data confidentiality.

Consistent with the observational nature of this study, no additional interventions, investigations, or procedures were performed for research purposes. The 75 g oral glucose tolerance test (OGTT) administered to all participants represents standard antenatal care recommended at 24–28 gestational weeks by the World Health Organization, the Turkish Ministry of Health, and international obstetric societies including the International Association of Diabetes and Pregnancy Study Groups [2] and the American College of Obstetricians and Gynecologists [4]. All OGTT testing was provided under Turkish Ministry of Health social security coverage and routine institutional services; participation in the study imposed no additional cost, procedure, or risk on participants. The research protocol comprised only the prospective documentation and analysis of anthropometric and laboratory data that would have been obtained as part of routine clinical care, irrespective of study enrollment.

### 2.2. Study Population

Between 9 February and 19 February 2026, we prospectively enrolled consecutive pregnant women presenting to the obstetric outpatient clinic of Ankara Etlik City Hospital who met the predefined eligibility criteria. Ankara Etlik City Hospital is one of the largest tertiary referral centers in Türkiye. During the enrollment period, obstetric outpatient care was delivered through eleven parallel general obstetric clinics operating on every working day. According to institutional outpatient records, a total of 4284 pregnant women were examined across these clinics over the enrollment window (9–19 February 2026), corresponding to an average of approximately 476 pregnant women per working day. Screening was performed prospectively and consecutively: each pregnant woman presenting to any of these clinics was assessed against the predefined eligibility criteria at the point of her first-trimester visit, and those who met all criteria were enrolled. Against this screening denominator of 4284 women, the 198 participants who satisfied the full set of restrictive inclusion and exclusion criteria represented approximately 4.6% of all pregnant women examined—an inclusion rate that is fully consistent with prospective, real-time recruitment given the deliberately narrow eligibility window, and that accounts for the cohort size accrued over this period. The study population was deliberately defined a priori to address a specific clinical gray zone within current screening guidelines: overweight pregnant women who do not carry established major risk factors for gestational diabetes mellitus (GDM), and for whom existing recommendations provide no clear directive regarding early screening [4,5].

Inclusion criteria were as follows: women aged 18 to 45 years; singleton pregnancy; body mass index (BMI) of 25.0 to 29.9 kg/m^2^ (overweight category per World Health Organization classification); first antenatal visit between 11 + 0 and 13 + 6 gestational weeks; and provision of written informed consent.

Exclusion criteria were as follows: pre-gestational diabetes mellitus (type 1 or type 2); first-degree family history of diabetes; previous gestational diabetes; polycystic ovary syndrome; previous macrosomic infant (birth weight > 4000 g); multiple pregnancy; and diagnosis of diabetes before 24 gestational weeks. At Ankara Etlik City Hospital, obstetric and gynecologic clinics are structurally separated, with polycystic ovary syndrome and other gynecologic endocrine conditions managed in dedicated gynecology and reproductive endocrinology services, and high-risk pregnancies followed in a separate perinatology clinic. Polycystic ovary syndrome was ascertained on the basis of a documented prior diagnosis (Rotterdam criteria) in the integrated medical record. Other endocrine conditions with potential metabolic relevance, including untreated thyroid dysfunction, were identified and excluded through review of clinical history and the routine first-trimester laboratory panel, which included thyroid function testing (TSH, free T3, free T4). Eligibility was assessed at enrollment through structured clinical history and review of the integrated electronic medical record; any participant identified as high-risk during this assessment was referred to perinatology and excluded from the study cohort.

A total of 198 pregnant women meeting the eligibility criteria were enrolled. All enrolled participants underwent standardized anthropometric assessment during the first trimester (Section 2.3) and were scheduled for the routine 75 g OGTT at 24–28 gestational weeks. At 24–28 gestational weeks (the OGTT screening window), completion status of the OGTT was verified through medical record review prior to assessment of any glucose value, ensuring that exclusion decisions could not be influenced by knowledge of GDM status. This verification identified 12 participants who did not contribute outcome data: two women transferred their antenatal follow-up to another institution and discontinued care at our center, and ten women declined the scheduled OGTT based on personal preference despite physician recommendation. These 12 participants were excluded from the analysis. Consequently, the analytic cohort comprised 186 pregnant women, corresponding to the a priori planned sample size (Section 2.6).

### 2.3. Anthropometric Measurements

All anthropometric measurements were performed during the first antenatal visit (11 + 0 to 13 + 6 gestational weeks) in a dedicated anthropometry room within the obstetric outpatient clinic. Measurements were obtained by two trained registered nurses who had received standardized training on the measurement protocol prior to study initiation. Each nurse independently performed two measurements per participant, yielding four measurements per anthropometric variable; the arithmetic mean of these four measurements was used in all subsequent analyses to minimize measurement variability. Although individual measurement values were not retained in the database for post hoc reliability analysis, the use of four independent measurements with averaging was adopted prospectively to reduce measurement-related variance, providing a variance-reduction benefit comparable to that achieved through formal reliability-corrected approaches.

Height was measured to the nearest 0.1 cm using a calibrated wall-mounted stadiometer (Seca GmbH, Hamburg, Germany), with participants standing barefoot, heels together, and head positioned in the Frankfurt horizontal plane. Body weight was measured to the nearest 0.1 kg using a calibrated digital scale (Seca GmbH, Hamburg, Germany), with participants wearing light clothing and no shoes. Waist circumference was measured to the nearest 0.1 cm using a non-elastic measuring tape (Seca GmbH, Hamburg, Germany) at the midpoint between the lower costal margin and the iliac crest, with the participant in a standing position at the end of normal expiration. The first-trimester time window (11 + 0 to 13 + 6 weeks) was selected because the gravid uterus remains confined within the pelvic cavity during this period, minimizing potential confounding of waist circumference by uterine enlargement.

Body mass index (BMI) was calculated as weight in kilograms divided by the square of height in meters (kg/m^2^). The Body Roundness Index (BRI) was calculated according to the formula proposed by Thomas et al. [8]:

BRI = 364.2 − 365.5 × √(1 − [(WC/2π)^2^/(0.5 × height)^2^])

where WC denotes waist circumference and height is expressed in the same units. A Body Shape Index (ABSI) was calculated according to the formula proposed by Krakauer and Krakauer [9]:

ABSI = WC/(BMI^(2/3)^ × height^(1/2)^)

with WC and height in meters and BMI in kg/m^2^.

### 2.4. OGTT and Laboratory Analysis

The 75 g oral glucose tolerance test (OGTT) was performed between 24 + 0 and 28 + 0 gestational weeks following the IADPSG protocol [2]. Participants were instructed to maintain their usual diet and physical activity for at least three days prior to testing and to fast for a minimum of 8 h before the test. Fasting status was confirmed by direct questioning at the time of blood sampling. A fasting venous blood sample was obtained, after which participants ingested a standardized 75 g anhydrous glucose solution dissolved in 300 mL of water within five minutes. Additional venous blood samples were collected at 1 h and 2 h after glucose ingestion. Participants remained seated and refrained from eating, drinking, smoking, or strenuous activity throughout the test.

All blood samples were collected into serum separator tubes (BD Vacutainer, Becton, Dickinson and Company, Franklin Lakes, NJ, USA) and processed at the Department of Medical Biochemistry, Ankara Etlik City Hospital. Serum glucose concentrations were measured using a hexokinase-based enzymatic reference method on a Roche Cobas 8000 c702 automated biochemistry analyzer (Roche Diagnostics, Mannheim, Germany). The laboratory operates in accordance with the quality standards of the Turkish Ministry of Health, with an intra-assay coefficient of variation for the glucose assay verified at ≤1.5%. Analyzer calibration was performed weekly and monthly as part of routine quality assurance, with additional calibration triggered automatically by quality control alerts; internal quality control procedures were conducted every 12 h.

All OGTT testing was provided under Turkish Ministry of Health social security coverage and routine institutional services, and an identical pre-analytical and analytical protocol was applied to every participant throughout the study period, ensuring homogeneity of testing conditions across the cohort.

### 2.5. GDM Diagnosis

Gestational diabetes mellitus was diagnosed according to the criteria established by the International Association of Diabetes and Pregnancy Study Groups [2]. Following the one-step 75 g OGTT, a diagnosis of GDM was made when one or more of the following plasma glucose threshold values were met or exceeded: fasting plasma glucose ≥ 92 mg/dL (5.1 mmol/L), 1 h plasma glucose ≥ 180 mg/dL (10.0 mmol/L), or 2 h plasma glucose ≥ 153 mg/dL (8.5 mmol/L).

OGTT results were independently reviewed by attending obstetricians, who were not involved in the first-trimester anthropometric assessment. Conversely, the trained nurses who performed the first-trimester anthropometric measurements had no access to subsequent OGTT outcomes, ensuring that anthropometric data collection was performed blinded to the eventual GDM status. Participants meeting at least one diagnostic threshold were classified as GDM-positive; those whose values remained below all three thresholds were classified as GDM-negative.

### 2.6. Statistical Analysis

Sample Size Determination: The required sample size was determined a priori through power analysis using G*Power 3.1 (Heinrich Heine University, Düsseldorf, Germany). The analysis assumed a two-tailed binary logistic regression model with α = 0.05 and statistical power (1 − β) = 0.95. Based on literature evidence available at the time of protocol development, the anticipated effect size was set at an odds ratio of 5.71, with a conservative baseline event probability of 0.03 in the reference (unexposed) group (Pr[Y = 1|X = 0] = 0.03) and an R^2^-other-X value of 0 to reflect the primary single-predictor framework. This conservative baseline was deliberately adopted to maximize the required sample size and ensure adequate power under low event-rate assumptions; the observed cohort GDM prevalence of 18.28% substantially exceeded this conservative protocol-stage assumption, providing additional power margin in practice. Under these parameters, the required minimum sample size was calculated as 186 participants; to account for potential attrition and incomplete records, enrollment of approximately 200 participants was planned. The final analytic cohort (*n* = 186) corresponded precisely to the a priori planned sample size. The conservative power assumption (1 − β = 0.95) was deliberately adopted to ensure adequate power even under modest anticipated effect sizes; the effect sizes ultimately observed substantially exceeded these a priori assumptions.

Software: All statistical analyses were performed using IBM SPSS Statistics version 27.0 (IBM Corp., Armonk, NY, USA) and R version 4.3.0 (R Foundation for Statistical Computing, Vienna, Austria). Descriptive statistics, group comparisons, ROC analyses, and logistic regression models were performed in SPSS. Comparison of ROC curves using DeLong’s test, bootstrap optimism correction, k-fold and leave-one-out cross-validation, calibration analysis, decision curve analysis, and reclassification metrics were performed in R using the pROC, rms, ResourceSelection, rmda, and PredictABEL packages.

Descriptive and Comparative Statistics: Continuous variables were assessed for normality using the Shapiro–Wilk test and visual inspection of distributions. Normally distributed variables are presented as mean ± standard deviation and compared using the independent samples *t*-test; non-normally distributed variables are presented as median (interquartile range) and compared using the Mann–Whitney U test. Categorical variables are presented as frequencies and percentages and compared using the chi-square test or Fisher’s exact test, as appropriate. Effect sizes were calculated as Cohen’s *d* for parametric comparisons and *r* (=Z/√N) for non-parametric comparisons.

ROC Analysis: The discriminative performance of BMI, waist circumference, BRI, and ABSI for GDM was evaluated by receiver operating characteristic (ROC) curve analysis. Areas under the curve (AUC) are reported with 95% confidence intervals calculated using the method of Hanley and McNeil. Optimal cutoff values for each anthropometric index were derived using the Youden index (J = sensitivity + specificity − 1), and corresponding sensitivity, specificity, positive predictive value (PPV), negative predictive value (NPV), positive likelihood ratio (LR+), and negative likelihood ratio (LR−) were calculated. AUCs of competing predictors were compared using DeLong’s test for paired ROC curves [26].

Logistic Regression: Univariate logistic regression analyses were performed to estimate odds ratios (OR) with 95% confidence intervals for each candidate predictor. Standardized odds ratios per one standard deviation increment were also calculated to enable direct comparison of effect sizes across predictors with different measurement scales. Multivariate logistic regression models were constructed to evaluate the independent contributions of anthropometric indices after adjustment for age and BMI. Multicollinearity was assessed using the variance inflation factor (VIF), with VIF < 5 considered acceptable.

Model Performance and Internal Validation: Model calibration was evaluated using the Hosmer–Lemeshow goodness-of-fit test, calibration-in-the-large, apparent calibration slope, and the Brier score; the apparent calibration slope is, by definition, equal to 1.0 when computed on the same data used to fit the model and is reported here to document this property explicitly. A calibration plot for the primary model (Model A) is provided as Supplementary Figure S1. To assess the robustness of model performance and quantify potential optimism, three complementary internal validation procedures were applied: (i) bootstrap optimism correction using 1000 resamples, (ii) stratified 5-fold cross-validation, and (iii) leave-one-out cross-validation. The convergence of these methods was used as the principal indicator of internal validity.

Clinical Utility Analyses: The clinical utility of the candidate predictors was further evaluated using decision curve analysis (DCA), which quantifies net benefit across a range of threshold probabilities relative to “treat all” and “treat none” reference strategies. Reclassification improvement when adding BRI or ABSI to a BMI-only base model was quantified using the continuous net reclassification improvement (NRI) and integrated discrimination improvement (IDI). External applicability of a previously published BRI cutoff [14] was evaluated by applying it to our cohort and computing the corresponding diagnostic indices.

Subgroup Analyses: Pre-specified subgroup analyses were performed by stratifying the cohort into three predefined BMI subgroups based on clinically meaningful cut-points within the overweight range (25.0–25.99, 26.0–27.99, and 28.0–29.9 kg/m^2^) to explore variation in discriminative performance across the overweight spectrum. The reporting of diagnostic accuracy results follows the Standards for Reporting of Diagnostic Accuracy Studies (STARD) 2015 guidelines [27] (Table S1), and the development and reporting of the prediction models were additionally informed by the Transparent Reporting of a multivariable prediction model for Individual Prognosis Or Diagnosis (TRIPOD) recommendations [28].

Significance Threshold: A two-sided *p*-value < 0.05 was considered statistically significant throughout the analyses.

## 3. Results

### 3.1. Participant Flow and Final Analytic Cohort

**Figure 1:**
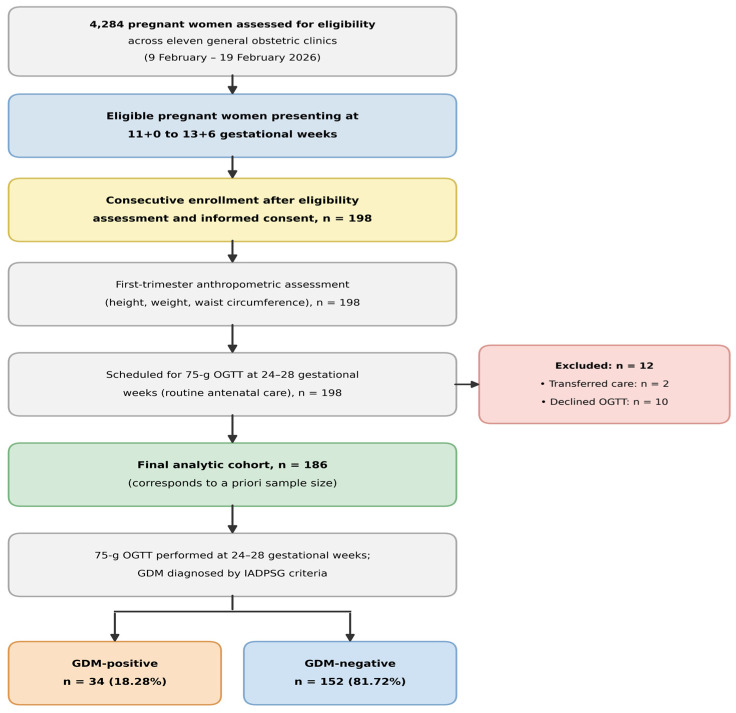
STARD flow diagram showing study recruitment, eligibility assessment, and final analytic cohort. STARD = Standards for Reporting of Diagnostic Accuracy Studies; OGTT = oral glucose tolerance test; GDM = gestational diabetes mellitus; IADPSG = International Association of Diabetes and Pregnancy Study Groups; GW = gestational weeks.

Of 198 consecutive eligible pregnant women who provided written informed consent and underwent first-trimester anthropometric assessment between 9 February and 19 February 2026, a total of 12 participants did not contribute outcome data (10 declined the scheduled 75 g OGTT and 2 transferred their antenatal follow-up to other institutions). The final analytic cohort therefore comprised 186 pregnant women, corresponding exactly to the a priori planned sample size derived from the G*Power power analysis (Section 2.6). Of these, 34 (18.28%) developed gestational diabetes mellitus by IADPSG criteria at 24–28 gestational weeks, and 152 (81.72%) did not. Participant flow is summarized in Figure 1.

### 3.2. Baseline Characteristics

Baseline demographic and anthropometric characteristics of the analytic cohort (*n* = 186) are summarized in Table 1. With the exception of maternal height, all continuous variables were non-normally distributed by the Shapiro–Wilk test and are therefore presented as median (interquartile range, IQR). The median maternal age was 27.0 years (IQR 25.0–32.0; range 18–41 years), and the median gestational age at first-trimester assessment was 12.43 weeks (IQR 12.00–13.00). Of the 186 participants, 78 (41.9%) were nulliparous and 108 (58.1%) were multiparous, with a median gravida of 2 (IQR 1–3) and a median parity of 1 (IQR 0–1).

Mean maternal height was 162.41 ± 6.48 cm. Median body weight was 71.0 kg (IQR 67.0–77.0). By design, the BMI distribution was confined to the overweight range, with a median of 26.86 kg/m^2^ (IQR 25.46–29.12) and a full range of 25.00–29.78 kg/m^2^. Median waist circumference was 85.0 cm (IQR 80.0–89.0). The derived anthropometric indices were a median BRI of 3.77 (IQR 3.24–4.34; range 1.83–7.08) and a median ABSI of 0.0737 (IQR 0.0707–0.0768; range 0.062–0.091), reflecting the expected distributions in an overweight pregnant cohort at 11 + 0 to 13 + 6 gestational weeks.

### 3.3. Comparison of GDM-Positive and GDM-Negative Groups

A comparison of baseline and anthropometric variables between the GDM-positive (*n* = 34) and GDM-negative (*n* = 152) groups is presented in Table 2. Variables were tested for normality within each group and compared using the appropriate parametric or non-parametric test (Methods, Section 2.6). The two groups did not differ significantly in age (median 28.5 [IQR 25.0–33.0] vs. 27.0 [IQR 25.0–32.0] years; *p* = 0.255), gestational age at first-trimester assessment (median 12.36 vs. 12.43 weeks; *p* = 0.792), gravida (*p* = 0.444), parity (*p* = 0.236), maternal height (median 160.0 vs. 162.0 cm; *p* = 0.113), or maternal weight (median 74.0 vs. 71.0 kg; *p* = 0.228). The proportion of nulliparous women was also comparable between groups (35.3% vs. 43.4%; chi-square *p* = 0.499).

In contrast, all four primary anthropometric measures differed substantially between groups. Median BMI was higher in the GDM-positive group (28.91 vs. 26.47 kg/m^2^; Mann–Whitney *p* < 0.001; effect size *r* = 0.27), as was median waist circumference (95.0 vs. 83.5 cm; *p* < 0.001; *r* = 0.49). The magnitude of group separation was greatest for the derived shape indices, both of which were normally distributed within each outcome group and were therefore compared using the independent samples t-test: mean BRI was 5.17 ± 0.98 in the GDM-positive group versus 3.60 ± 0.72 in the GDM-negative group (*p* < 0.001; Cohen’s *d* = 2.04), and mean ABSI values were 0.080 ± 0.005 versus 0.072 ± 0.004, respectively (*p* < 0.001; Cohen’s *d* = 1.83). The considerably larger standardized mean differences observed for BRI and ABSI compared with BMI suggest that these composite indices capture additional information related to GDM risk beyond what is reflected by BMI alone in this overweight cohort.

### 3.4. ROC Analysis and Diagnostic Performance

**Figure 2:**
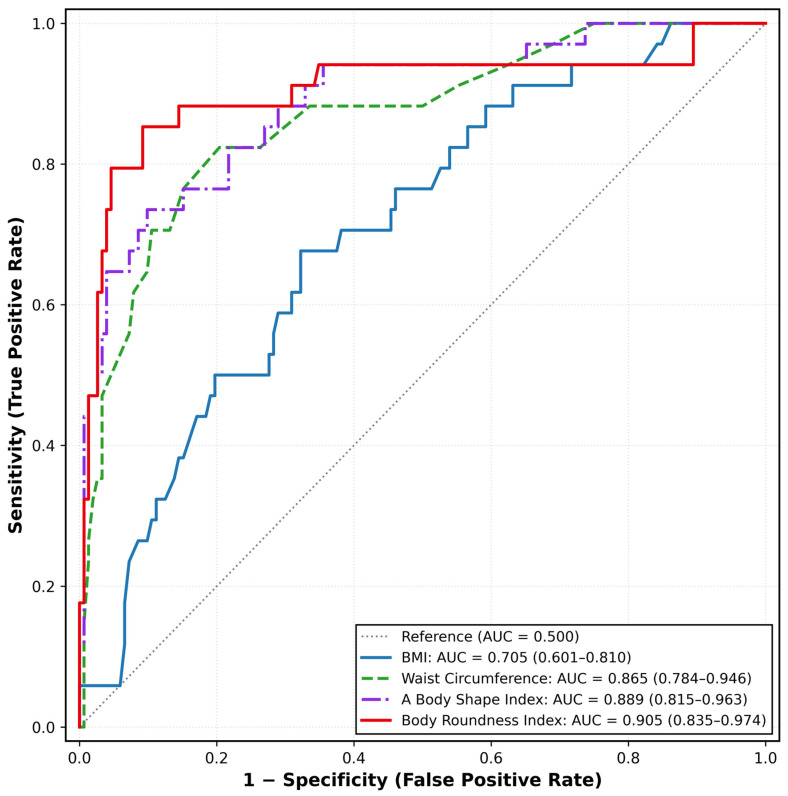
Receiver operating characteristic (ROC) curves for first-trimester body mass index (BMI), waist circumference, A Body Shape Index (ABSI), and Body Roundness Index (BRI) in predicting subsequent IADPSG-defined gestational diabetes mellitus (*n* = 186). Area under the curve (AUC) values are shown in the legend with 95% confidence intervals (Hanley–McNeil method).

Receiver operating characteristic (ROC) analyses for prediction of subsequent GDM are presented in Table 3 and shown graphically in Figure 2. Body mass index showed moderate discriminative ability (AUC 0.705; 95% CI 0.601–0.810), whereas all three waist-based measures performed substantially better: waist circumference (AUC 0.865; 95% CI 0.784–0.946), A Body Shape Index (AUC 0.889; 95% CI 0.815–0.963), and Body Roundness Index (AUC 0.905; 95% CI 0.835–0.974).

The Youden-derived optimal cutoff for BRI was 4.45, with a sensitivity of 85.3%, specificity of 90.8%, positive predictive value of 67.4%, negative predictive value of 96.5%, positive likelihood ratio of 9.26, and negative likelihood ratio of 0.16. The corresponding optimal cutoff for ABSI was 0.0772 (sensitivity 73.5%, specificity 90.1%, PPV 62.5%, NPV 93.8%, LR+ 7.45, LR− 0.29). For comparison, the optimal cutoff for BMI alone was 28.04 kg/m^2^ (sensitivity 67.6%, specificity 67.8%, PPV 31.9%, NPV 90.4%), and for waist circumference, 88.0 cm (sensitivity 82.4%, specificity 79.6%, PPV 47.5%, NPV 95.3%).

Pairwise comparison of AUCs using DeLong’s test confirmed that BRI, ABSI, and waist circumference each provided significantly greater discrimination than BMI (BRI vs. BMI: ΔAUC = +0.199, *p* < 0.001; ABSI vs. BMI: ΔAUC = +0.184, *p* < 0.001; waist circumference vs. BMI: ΔAUC = +0.159, *p* < 0.001). Differences between BRI and ABSI (ΔAUC = +0.016, *p* = 0.462), between BRI and waist circumference (ΔAUC = +0.040, *p* = 0.071), and between ABSI and waist circumference (ΔAUC = +0.024, *p* = 0.124) were not statistically significant, indicating that the three central-adiposity measures provide comparable discriminative performance in this cohort. Although BRI and ABSI yielded statistically comparable AUC values, the two indices are constructed to capture complementary aspects of body composition: BRI integrates waist circumference and height in an ellipse-based geometric model that approximates overall body roundness, whereas ABSI isolates the shape-specific component of waist circumference that is independent of body mass index. This distinction is reflected in their differing operating characteristics at the Youden-derived cutoffs, with BRI achieving higher sensitivity (85.3% versus 73.5%) at comparable specificity.

### 3.5. Logistic Regression Analyses

Univariate and multivariate logistic regression analyses were performed to quantify the strength of association between each anthropometric predictor and GDM. Because the four anthropometric variables are measured on different scales, standardized odds ratios per one standard deviation (SD) increment are also reported to enable direct comparison of effect sizes. Detailed regression results are presented in Table 4.

In univariate analyses, maternal age was not significantly associated with GDM (OR per 1 SD = 1.29; 95% CI 0.89–1.87; *p* = 0.177). All four anthropometric variables were significantly associated with GDM, with effect sizes increasing along the BMI → WC → ABSI → BRI sequence. The standardized odds ratios per 1 SD increment were 2.08 (95% CI 1.38–3.14; *p* < 0.001) for BMI, 5.42 (95% CI 3.09–9.51; *p* < 0.001) for waist circumference, 7.91 (95% CI 3.92–15.97; *p* < 0.001) for ABSI, and 9.52 (95% CI 4.63–19.56; *p* < 0.001) for BRI. The substantially larger standardized odds ratios for BRI and ABSI compared with BMI are consistent with the discrimination differences observed in the ROC analysis.

Two multivariate models were considered. Model A combined maternal age and BRI, both expressed per 1 SD increment. In this model, BRI remained strongly associated with GDM (OR per 1 SD = 9.44; 95% CI 4.56–19.54; *p* < 0.001), whereas age was not significant (OR per 1 SD = 1.04; 95% CI 0.64–1.69; *p* = 0.885). Multicollinearity was not a concern (variance inflation factor [VIF] = 1.02 for both variables). The model achieved an AUC of 0.9044 with a pseudo-R^2^ of 0.4418. Model A was specified as a parsimonious, age-adjusted BRI model. BMI and BRI were not entered simultaneously into this primary association model because their joint inclusion produced unstable, sign-reversed coefficients consistent with statistical suppression, reflecting substantial empirical correlation between the two measures in this cohort. The incremental value of combining BMI with a shape-based index was instead evaluated separately through the reclassification and decision-curve analyses (Section 3.7).

Model B combined maternal age, BMI, and ABSI, all expressed per 1 SD increment, reflecting the fact that ABSI is, by construction, designed to be largely independent of BMI. In this model, both BMI (OR per 1 SD = 1.81; 95% CI 1.07–3.05; *p* = 0.028) and ABSI (OR per 1 SD = 7.13; 95% CI 3.49–14.54; *p* < 0.001) were independently associated with GDM, while age remained non-significant (OR per 1 SD = 1.02; 95% CI 0.61–1.68; *p* = 0.863). All variance inflation factors were below 2.0 (age: 1.04; BMI: 1.08; ABSI: 1.11), indicating an absence of multicollinearity with BMI in this cohort, consistent with the index’s mathematical construction. The model achieved an AUC of 0.8996 with a pseudo-R^2^ of 0.4116.

Taken together, the univariate and multivariate analyses indicate that BRI and ABSI each carry strong independent information about GDM risk in overweight pregnant women without major established risk factors, with comparable performance to one another and consistently stronger associations than BMI alone.

### 3.6. Calibration and Internal Validation

Calibration was evaluated using the Hosmer–Lemeshow goodness-of-fit test, calibration-in-the-large, and the Brier score. Calibration-in-the-large was 0.000 for all models, indicating no systematic over- or under-prediction on average. Brier scores were low across all candidate predictors (BRI: 0.072; ABSI: 0.086; Model A: 0.071; Model B: 0.079), with lower values indicating better calibration. The Hosmer–Lemeshow test yielded mixed results: it was significant for BRI-based models (BRI univariate: χ^2^ = 72.04, *p* < 0.001; Model A: χ^2^ = 71.22, *p* < 0.001) and for Model B (χ^2^ = 18.81, *p* = 0.016), but non-significant for ABSI alone (χ^2^ = 10.50, *p* = 0.232). This represents a discrimination–calibration dissociation, in which discriminative performance is strong but absolute predicted probabilities deviate from observed frequencies in some risk strata; this is a recognized phenomenon when a strong predictor is applied without explicit recalibration. The significant Hosmer–Lemeshow result for BRI-based models indicates that absolute predicted probabilities deviate from observed frequencies in some risk strata, and should be interpreted alongside two considerations: first, the concentration of predicted probabilities toward the extremes of the risk distribution, which can accentuate apparent miscalibration when a strong predictor is modeled without explicit recalibration; and second, the well-documented sensitivity of the Hosmer–Lemeshow statistic to sample size and to the arbitrary grouping of predicted risks. We therefore interpret this finding as an indication that the model requires recalibration before use for absolute risk estimation, rather than as evidence against its discriminative validity. The clinical implication is that the predicted probabilities derived from this cohort would require recalibration before application to populations with a different baseline GDM prevalence.

Internal validity of model performance was further evaluated through three complementary procedures (Table 5). Bootstrap optimism correction (1000 resamples) demonstrated minimal optimism in all models: BRI (apparent 0.9047 → corrected 0.9041, optimism +0.0006), ABSI (apparent 0.8891 → corrected 0.8908, optimism −0.0017), Model A (apparent 0.9044 → corrected 0.9022, optimism +0.0022), and Model B (apparent 0.8996 → corrected 0.8929, optimism +0.0066). Stratified 5-fold cross-validation produced mean AUC values of 0.8993 (±0.0438) for BRI, 0.8913 (±0.0460) for ABSI, 0.8961 (±0.0469) for Model A, and 0.8812 (±0.0452) for Model B. Leave-one-out cross-validation yielded AUCs of 0.8973 for BRI, 0.8796 for ABSI, 0.8953 for Model A, and 0.8849 for Model B. The close convergence of these three independent validation procedures, with optimism remaining below 0.01 across all models, indicates that the observed discriminative performance is unlikely to reflect substantial overfitting within this cohort.

### 3.7. Decision Curve Analysis, Reclassification, and External Cutoff Application

**Figure 3:**
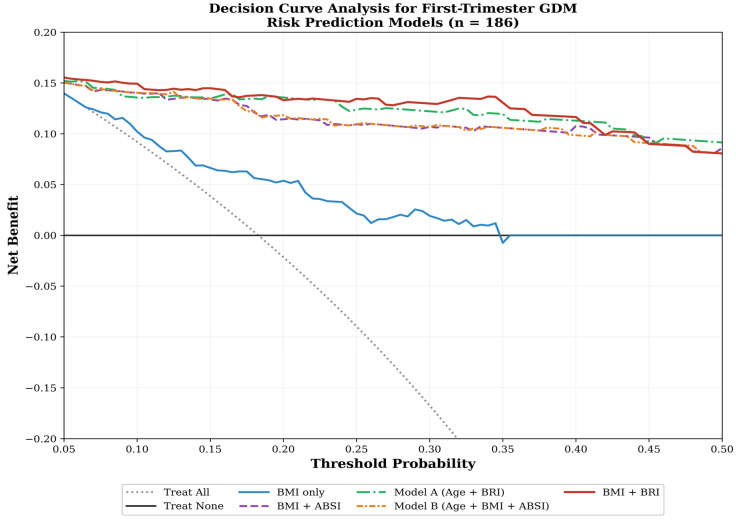
Decision curve analysis comparing net benefit across threshold probabilities for first-trimester gestational diabetes mellitus risk prediction models. Reference strategies are “Treat All” (dotted gray) and “Treat None” (solid black). Across the clinically relevant threshold range, Model A (Age + BRI) and BMI + BRI provide closely overlapping curves with the highest net benefit, both clearly dominating BMI alone and the “Treat All” strategy.

Decision curve analysis was performed to evaluate the clinical net benefit of using anthropometric indices for first-trimester GDM risk stratification across a clinically relevant range of threshold probabilities (Figure 3). At a threshold probability of 0.20—selected as clinically representative for first-trimester GDM risk assessment, corresponding approximately to a number needed to investigate of 5 and lying just above the baseline cohort prevalence of 18.28%—the net benefit of a “treat all” strategy was −0.022, while a BMI-only model provided a net benefit of +0.054. Adding BRI to BMI substantially increased net benefit to +0.133, and adding ABSI to BMI increased it to +0.114. Model A (Age + BRI) yielded the highest net benefit of +0.136, with Model B (Age + BMI + ABSI) yielding +0.118. Across the clinically relevant threshold range of 0.10 to 0.40, the BMI + BRI combination consistently demonstrated the highest net benefit, dominating both BMI alone and “treat all” reference strategies.

The incremental value of adding composite anthropometric indices to a BMI-only base model was quantified through reclassification metrics. Adding BRI to the BMI base model produced an events net reclassification improvement (NRI) of +0.706 and a non-events NRI of +0.645 (continuous NRI = +1.351), with an integrated discrimination improvement (IDI) of +0.456 (+45.6%). Adding ABSI to the BMI base model produced an events NRI of +0.529 and a non-events NRI of +0.592 (continuous NRI = +1.122), with an IDI of +0.383 (+38.3%). These results indicate that incorporation of either BRI or ABSI improves risk classification beyond what is achievable with BMI alone in this cohort. These reclassification metrics should, however, be interpreted with appropriate caution. The base model was deliberately restricted to BMI alone, and improvement statistics such as the NRI and IDI are known to yield large values when an informative predictor is added to a minimal reference model; the magnitude of the reported improvement is therefore conditional on this simple comparator and should not be read as an absolute measure of clinical gain. Furthermore, with 34 events, both indices are sensitive to the reclassification of a small number of individuals, so the point estimates reported here carry appreciable uncertainty and require confirmation in larger cohorts.

We additionally examined the transportability of a previously published BRI cutoff. A BRI cutoff of 6.708 was previously proposed in a Turkish midgestation cohort [14]. Applying this external cutoff to our first-trimester cohort identified only 2 of 186 participants (1.1%) as above threshold, yielding a sensitivity of 5.9% and specificity of 100%. This trimester-shift in operating characteristics—high specificity at the cost of severely reduced sensitivity—reflects the differing distributions of BRI between first-trimester (median 3.77) and mid-trimester (where BRI values are systematically higher due to increased waist circumference from uterine enlargement) populations. The finding supports the use of a cohort-specific cutoff (4.45) for first-trimester application and underscores the importance of population-specific calibration before any cross-population deployment. Importantly, this argument is symmetrical: just as the previously published cutoff of 6.708 failed to transport to our first-trimester cohort, our own cutoff of 4.45 cannot be assumed to perform reliably in populations that differ in gestational-age window, ethnicity, or baseline risk distribution. The 4.45 threshold should therefore be interpreted strictly as an analytic anchor derived from and applicable to this specific cohort, and its transportability to other settings must itself be established through external validation rather than presumed.

### 3.8. Subgroup Analyses by Predefined BMI Subgroups

Pre-specified subgroup analyses were performed to explore variation in discriminative performance across the overweight spectrum. The cohort was stratified into three predefined BMI subgroups based on clinically meaningful cut-points within the overweight range: lower (25.0–25.99 kg/m^2^; *n* = 69), middle (26.0–27.99 kg/m^2^; *n* = 43), and upper (28.0–29.9 kg/m^2^; *n* = 74). These subgroups were defined a priori for descriptive stratification across the overweight range and do not represent statistical tertiles of the BMI distribution. GDM prevalence increased markedly across BMI subgroups, from 7.2% (5/69) in the lower subgroup to 14.0% (6/43) in the middle subgroup and 31.1% (23/74) in the upper subgroup, with the upper subgroup accounting for 67.6% (23/34) of all GDM events in the cohort.

Discriminative performance of BRI varied across subgroups: AUC was 0.678 (95% CI 0.410–0.946) in the lower BMI subgroup, 0.946 in the middle BMI subgroup, and 0.936 in the upper BMI subgroup. The wide confidence interval in the lower subgroup (which contained only 5 GDM events) precludes firm conclusions about discriminative performance at the lower end of the overweight range; however, the consistency of strong performance in the middle and upper subgroups (which together contained 29 of 34 GDM events) supports the robustness of the overall cohort-level findings. These subgroup results are reported transparently here and were interpreted as exploratory rather than confirmatory, as our a priori power calculation was designed to detect the overall primary effect rather than subgroup-specific differences.

## 4. Discussion

In this prospective single-center cohort study of 186 overweight pregnant women without established major risk factors for gestational diabetes mellitus, both Body Roundness Index and A Body Shape Index measured between 11 + 0 and 13 + 6 gestational weeks demonstrated strong discriminative performance for subsequent IADPSG-defined GDM, with AUC values of 0.905 (95% CI 0.835–0.974) for BRI and 0.889 (95% CI 0.815–0.963) for ABSI. Both indices markedly outperformed BMI alone (AUC 0.705), with DeLong’s test confirming statistically significant differences (*p* < 0.001 for both pairwise comparisons). The optimal BRI cutoff of 4.45 yielded a sensitivity of 85.3%, specificity of 90.8%, and positive likelihood ratio of 9.26, while the optimal ABSI cutoff of 0.0772 yielded a sensitivity of 73.5% and specificity of 90.1%. Reclassification analyses demonstrated that adding BRI to a BMI-only base model improved discrimination by an IDI of 45.6%, and decision curve analysis confirmed a clinically meaningful net benefit across the relevant threshold range. Internal validity was confirmed through three independent validation procedures (bootstrap optimism correction, stratified 5-fold cross-validation, and leave-one-out cross-validation), whose close convergence (optimism < 0.01) indicates that the observed performance is unlikely to reflect substantial overfitting within this cohort. This convergence, however, does not substitute for prospective external validation, which remains essential before any clinical application.

The clinical significance of these findings rests not only on the magnitude of the discriminative performance observed but also on the specific population studied. We deliberately defined our cohort to capture a guideline gray zone that is rarely the explicit focus of GDM screening research: pregnant women who are overweight (BMI 25.0–29.9 kg/m^2^) but who do not carry any of the established major risk factors—prior GDM, family history of diabetes, polycystic ovary syndrome, prior macrosomia, or diabetes detected before 24 gestational weeks—on which current early-screening recommendations are anchored [5,6]. In this population, ACOG guidance does not endorse early screening on the basis of overweight status alone [4], ADA criteria do not formally apply, and the TOBOGM evidence base does not directly extend. Yet GDM prevalence in our cohort was 18.28%—comparable to the upper end of contemporary Turkish population estimates [3]—indicating that this subgroup carries a clinically meaningful risk burden that is not currently addressed by any explicit screening directive. This figure aligns with meta-analytic estimates of GDM prevalence in overweight and obese pregnant women (pooled prevalence 23%, 95% CI 20.2–25.9%) and indeed sits toward the lower end of that range—an expected position given our deliberate exclusion of obese women, who carry the highest adiposity-related risk, as well as women with established major risk factors [29]. The observation that a clinically meaningful prevalence persists even after excluding conventional risk factors suggests that overweight status may contribute to residual metabolic risk and merits further investigation as a component of first-trimester risk stratification. Should these findings be confirmed in external populations, a reliable first-trimester risk-stratification tool calibrated to this specific subgroup could, in principle, complement universal second-trimester OGTT screening by flagging women who might warrant closer follow-up—potential applications that would require prospective validation before any clinical adoption and that would in no way alter the universal screening recommendation, which remains the cornerstone of GDM detection.

We acknowledge that the report by Pape et al. [25] is currently available as a conference abstract; nonetheless, its multicenter design, large sample size (*n* = 9675), and prospective enrollment within the well-established NuMoM2b cohort confer methodological weight that merits direct discussion despite its preliminary publication format. Our discriminative findings stand in apparent contrast to those recently reported by Pape et al. [25] in the large multicenter NuMoM2b cohort (*n* = 9675 nulliparous pregnant women), which reported a first-trimester BRI AUC of 0.69 for GDM prediction—only modestly better than BMI (AUC 0.66)—and concluded that BRI did not provide substantive clinical advantage over BMI in their unselected population. In the overweight subgroup of NuMoM2b (*n* = 2410), the adjusted odds ratio for BRI ≥ 3.65 was 1.76 (95% CI 1.08–2.87), considerably more modest than the standardized odds ratios observed in our cohort. We consider this contrast informative rather than contradictory. Three differences in cohort composition and analytic framing offer a coherent explanation. First, NuMoM2b is an unselected nulliparous cohort spanning the full range of pre-pregnancy BMI categories, including underweight, normal-weight, overweight, and obese strata, in which much of the variance in metabolic risk is already captured by BMI itself. In such a cohort, a shape-based index will provide limited incremental information over BMI. Our cohort, by contrast, was restricted a priori to the overweight range and excluded women with established major GDM risk factors, focusing specifically on the variance in body composition that BMI alone cannot resolve. Second, the analytic comparator differs: NuMoM2b primarily contrasted BRI with BMI in a population where both predictors are confounded by overall body size, whereas our analyses compared anthropometric indices in a deliberately narrow BMI range where shape and central distribution emerge as the dominant axis of metabolic variation. Third, ethnic composition, healthcare-system context, and OGTT protocol differ between the two populations. Taken together, these considerations suggest that BRI may have limited generalized value across unselected obstetric populations but may retain meaningful incremental utility within specific clinical niches where BMI is, by design, an insufficient discriminator. The convergent and divergent aspects of our findings with respect to Pape et al. [25] should both be acknowledged: the present study does not contest their broader conclusion about BRI in unselected populations but, rather, illustrates that the operating characteristics of composite anthropometric indices are conditional on the population in which they are evaluated.

The biological plausibility of our findings can be situated within established mechanisms of GDM pathophysiology. Insulin resistance in pregnancy reflects a dynamic interplay between placental hormonal milieu and maternal adipose tissue, and visceral adipose tissue exerts a disproportionately greater contribution to systemic insulin resistance than subcutaneous adipose tissue through enhanced lipolysis, free fatty acid release, and secretion of pro-inflammatory adipokines [7]. Because BMI integrates only weight and height, two women with identical BMI values can differ considerably in the proportion and anatomical distribution of their adipose tissue—particularly in the ratio of central (visceral and abdominal subcutaneous) to peripheral fat depots. This anatomic distinction has direct metabolic relevance: at any given BMI, a more centrally distributed adiposity phenotype is associated with greater insulin resistance and higher GDM risk. By design, the prospective measurement of anthropometric indices at 11 + 0 to 13 + 6 gestational weeks established temporal precedence over the GDM diagnosis at 24–28 weeks; reverse causality is therefore unlikely to explain the observed associations.

Composite anthropometric indices that incorporate waist circumference are designed to capture precisely this dimension. BRI, derived from a geometric ellipse model parameterized by waist circumference relative to height, quantifies overall body roundness and correlates with measures of central adiposity obtained from imaging techniques such as dual-energy X-ray absorptiometry [8,10]. ABSI, by mathematical construction, isolates the component of waist circumference that is not explained by height and weight, providing a shape-specific signal that is largely independent of overall body size [9]. In our cohort, ABSI showed a variance inflation factor of 1.11 when adjusted simultaneously for age and BMI in Model B, with corresponding VIF values of 1.04 for age and 1.08 for BMI. The fact that all VIFs in Model B remained below 2.0—including the low ABSI VIF—indicates an absence of multicollinearity with BMI in this cohort, consistent with the index’s mathematical construction, and is in keeping with the additional effect estimate that ABSI yielded in multivariate regression beyond that explained by BMI alone. Consistent with its design, ABSI is best understood not as a stand-alone diagnostic test for any single condition but as a body-size-independent shape descriptor that conveys its greatest value when interpreted alongside complementary anthropometric and clinical parameters; accordingly, in the present analysis ABSI contributed within a multivariable model (Model B) that also incorporated BMI, rather than as an isolated predictor.

A further question concerns whether the geometric complexity of BRI and ABSI is justified when waist circumference alone already performs well in this cohort (AUC 0.865). Three considerations support the added value of the composite indices. First, waist circumference is an absolute measurement that is not standardized for body frame, so an identical waist value carries different metabolic meaning in a tall versus a short woman; BRI and ABSI explicitly incorporate height, normalizing central adiposity to body frame and thereby capturing shape information that raw waist circumference cannot convey. Second, at their Youden-derived operating points the composite indices achieved a more favourable balance of sensitivity and specificity than waist circumference: BRI yielded 85.3% sensitivity with 90.8% specificity, compared with 82.4% sensitivity and 79.6% specificity for waist circumference, translating into a markedly higher positive likelihood ratio (9.26 versus 4.04) and thus greater ability to rule in elevated risk. Third, although the pairwise AUC difference between BRI and waist circumference did not reach conventional statistical significance (ΔAUC = +0.040, *p* = 0.071), the point estimate consistently favoured BRI and approached significance despite the limited number of events, a pattern compatible with a genuine but modest incremental signal that larger cohorts would be better powered to confirm. We therefore do not position BRI and ABSI as replacements for waist circumference, but rather as height-standardized refinements whose shape-based formulation offers conceptual and operating-characteristic advantages that merit evaluation in adequately powered external cohorts.

The marked discriminative leverage observed for BRI and ABSI within our overweight cohort may reflect the particular metabolic geometry of this population. In a population with a narrow BMI range, between-subject variation in overall body size is constrained, so the dominant axis of inter-individual difference shifts toward variation in body shape and fat distribution. This is precisely the dimension that composite indices are constructed to capture. By contrast, in cohorts spanning the full BMI range, much of the variance in metabolic risk is already absorbed by BMI itself, leaving less residual variance for shape-based indices to explain. This perspective provides a coherent mechanistic interpretation of why composite anthropometric indices may show greater incremental value in a BMI-restricted subpopulation than in unselected pregnancy cohorts—consistent with the divergent findings between our results and those reported by Pape et al. [25] in their broader cohort.

Our findings can also be positioned within the broader literature linking central adiposity to gestational metabolic risk. Prior cohort studies have consistently reported that centrally distributed fat, rather than overall body mass, is a principal driver of gestational insulin resistance: central obesity has been shown to mediate a substantial proportion of the association between adiposity and GDM [19], and composite obesity indices have outperformed BMI for GDM prediction across several populations [21]. Recent evidence extends this pattern to early pregnancy specifically, with early-gestation central-adiposity measures predicting both gestational diabetes and hypertensive disorders [13] and reinforcing the observation that BMI alone captures only part of the adiposity-related risk signal. Our results are concordant with the direction of these reports but add two elements that the existing literature has rarely addressed together: first, a deliberate restriction to the overweight, otherwise-low-risk stratum in which BMI is least informative; and second, the systematic comparison of two distinct shape-based indices (BRI and ABSI) against both BMI and waist circumference within the same first-trimester cohort. At the same time, the discriminative magnitude observed here exceeds that reported in most unselected cohorts, which we attribute—as discussed above—to the constrained BMI range of our population rather than to a fundamentally different biological effect; this interpretation, however, remains provisional until confirmed in external cohorts.

If our findings are externally validated, several practical implications for first-trimester antenatal care in the overweight low-risk subgroup could be envisaged. The required measurements—maternal height, weight, and waist circumference—are routinely obtainable during the initial obstetric visit at 11 + 0 to 13 + 6 weeks and impose no additional cost, equipment burden, or procedural risk on the patient. Computation of BRI and ABSI from these measurements is straightforward and can be readily incorporated into existing electronic medical record systems. In settings where such infrastructure is available, an early first-trimester anthropometric profile could serve as an adjunctive flag identifying overweight pregnant women without conventional risk factors who may benefit from intensified antenatal monitoring—for example, more frequent nutritional counseling, structured guidance on gestational weight gain, and earlier consideration of glucose surveillance in the late first or early second trimester.

We wish to emphasize, however, that the framing here is one of augmentation rather than replacement. The universal one-step 75 g OGTT at 24–28 gestational weeks remains the cornerstone of GDM diagnosis and is essential for all pregnant women regardless of any first-trimester risk-stratification approach. Composite anthropometric indices, even if validated in further studies, would function as supplementary risk-modifier tools that operate within the existing screening framework and do not displace it. For interpretive context, it is worth noting that BRI values have been divided into descriptive strata in the general (non-pregnant) population. These bands are most commonly derived as population quintiles from a large cohort of US adults, in which the lowest fifth corresponds to BRI < 3.41, the second to 3.41–4.45, the middle to 4.45–5.46, the fourth to 5.46–6.91, and the highest fifth to ≥6.91 [30]. It is important to emphasize that these are statistical population quintiles—not clinically validated diagnostic categories—and the accompanying descriptive labels (from lower to higher body roundness) denote relative position within that reference population rather than established risk thresholds. Two caveats are essential when relating these bands to our findings. First, these categories were established in non-pregnant populations and have not been validated for use in pregnancy, during which waist circumference and body composition change substantially; they cannot therefore be directly transposed to an obstetric cohort. Second, although our Youden-derived cutoff of 4.45 coincides numerically with the boundary between the “lean-to-average” and “average” bands in this general-population scheme, this correspondence is incidental and should not be interpreted as evidence that a general-population category boundary carries specific meaning for GDM risk. We include these descriptive bands solely to situate the observed BRI values within the broader anthropometric literature; the operative threshold in the present study remains the cohort-specific analytic anchor described below. Furthermore, the threshold-based clinical decisions that decision curve analysis explores are themselves contingent on local clinical priorities, available resources, and patient preferences; the BRI cutoff of 4.45 derived from our cohort should therefore be regarded as an analytic anchor for within-cohort interpretation rather than as a universally transferable clinical threshold. Until prospective external validation studies in independent populations confirm both the discriminative performance and the appropriate clinical thresholds for BRI and ABSI in the overweight low-risk niche, our findings should be regarded as hypothesis-generating—providing a structured rationale for larger multicenter investigations rather than a basis for immediate practice change.

Strengths: This study has several methodological strengths that warrant emphasis. First, the study population was defined a priori through deliberate restriction to overweight pregnant women without established major GDM risk factors, addressing a specifically identified gap in current screening guidelines. The cohort therefore represents a focused test of a pre-formulated hypothesis rather than a post hoc exploratory subgroup analysis. Second, the required sample size was calculated a priori using formal power analysis (G*Power 3.1) under explicit and conservative assumptions, and the final analytic cohort of 186 women corresponded precisely to this planned target. Third, the prospective consecutive enrollment design minimized selection bias within the eligibility window, and the integrated electronic medical record system permitted reliable verification of exclusion criteria at enrollment. Fourth, anthropometric assessment and GDM ascertainment were operationally separated: trained nurses performed first-trimester measurements blinded to subsequent OGTT results, while attending obstetricians independently evaluated OGTT outcomes without involvement in anthropometric data collection—a workflow that effectively eliminated differential measurement bias. Fifth, glucose measurements were performed using the hexokinase enzymatic reference method on a Roche Cobas 8000 c702 analyzer operating under Turkish Ministry of Health quality standards, with an intra-assay coefficient of variation ≤ 1.5%, calibration on weekly and monthly schedules supplemented by alert-triggered recalibration, and internal quality control performed every twelve hours. This places the laboratory pre-analytical and analytical environment at the upper end of clinically achievable precision. Sixth, anthropometric measurements were standardized by performing four measurements per variable—two independent measurements by each of two trained nurses—with the arithmetic mean used in all analyses, an approach designed to reduce measurement noise even in the absence of formal reliability coefficients. Seventh, internal validity of model performance was evaluated through three complementary procedures—bootstrap optimism correction, stratified k-fold cross-validation, and leave-one-out cross-validation—whose close convergence (optimism < 0.01 in all cases) provides cross-method evidence of robustness against overfitting within this cohort. Throughout the analysis, reporting was structured to conform with the Standards for Reporting of Diagnostic Accuracy Studies (STARD) 2015 framework [27].

Limitations: The findings should equally be interpreted in light of several limitations, which we discuss together with the design choices and mitigation strategies through which they were addressed.

First, the study was conducted at a single tertiary center in Ankara, Türkiye, and the cohort was drawn from a relatively homogeneous Turkish obstetric population. This homogeneity is methodologically advantageous for an initial hypothesis test, in which a controlled population framing isolates the signal of interest, but limits direct generalizability to populations with different ethnic backgrounds, body composition norms, or healthcare-system characteristics. We address this transparently by framing all numerical findings—including the BRI cutoff of 4.45—as within-cohort estimates explicitly awaiting external validation rather than as universally transferable thresholds. Relatedly, because the cohort was restricted a priori to the overweight BMI range, the diagnostic performance reported here reflects operating characteristics within this stratum and should not be extrapolated to unselected obstetric populations spanning the full BMI spectrum, in which the incremental value of shape-based indices over BMI is expected to be smaller.

Second, and most importantly, the absolute number of GDM events (*n* = 34) is modest and constitutes the principal limitation of this study. With only 34 events, the discriminative estimates reported here—most notably the BRI AUC of 0.905 and the standardized odds ratio of 9.52—should be regarded as optimistic within-cohort estimates that are likely to be attenuated in independent populations. It is essential to distinguish between two properties that our internal validation procedures address differently: the close convergence of bootstrap, k-fold, and leave-one-out estimates (optimism < 0.01) demonstrates that the models are numerically stable within this specific dataset, but such stability is not equivalent to external generalizability, which can only be established through prospective validation in independent cohorts. Moreover, cutoff-based performance is particularly fragile at this event count: the reported 85.3% sensitivity for BRI corresponds to correct classification of 29 of 34 events, so that reclassification of only a few individuals would materially shift the estimate. For the same reason, the absolute number of events also limits the precision of certain subgroup estimates, and confidence intervals for some AUC values, particularly within BMI subgroups, are correspondingly wide; the lower BMI subgroup contained only five GDM events, precluding firm conclusions about discriminative performance at the lower end of the overweight range. This constraint is inherent to the a priori power calculation, which was designed to detect the overall primary effect rather than subgroup-specific differences. To mitigate any inferential overreach, all subgroup analyses were pre-specified, reported transparently with their confidence intervals, and interpreted as exploratory rather than confirmatory. Additionally, baseline anthropometric data for the 12 participants who did not contribute outcome data could not be retrieved retrospectively, as ten women declined the OGTT and two had transferred their antenatal care to other centers, precluding a formal sensitivity analysis comparing included and excluded participants. We note, however, that the absolute number of excluded participants was small (6.1% of those initially enrolled) and that exclusion reasons were administrative rather than clinically related to the predictor variables of interest.

Third, although anthropometric assessment was performed as the mean of four independent measurements per participant, individual measurement values were not retained in the study database, so formal intraclass correlation coefficients to quantify inter- and intra-rater reliability could not be calculated post hoc. We mitigated this design feature prospectively through standardized training of the two nurses, use of a dedicated anthropometry room with calibrated equipment, and the deliberate use of multiple measurements with averaging, which together approximate the variance-reduction benefit that an explicit reliability analysis would have quantified. Future studies should retain individual measurements to permit explicit ICC reporting.

Fourth, although discriminative performance was strong and net benefit was clinically meaningful, the Hosmer–Lemeshow test was significant for BRI-based models. The Hosmer–Lemeshow statistic is well known to be sensitive to sample size and to flag deviations even when overall predictive accuracy is high; nevertheless, this finding correctly signals that absolute predicted probabilities derived from our regression coefficients should not be applied to external populations without prior recalibration, regardless of the strength of discrimination. We have therefore framed BRI and ABSI primarily as risk-stratification markers rather than as deployable risk-probability calculators, and the discrimination–calibration dissociation observed here is the subject of an explicit future-directions item below. The magnitude of this calibration pattern is illustrated in Supplementary Figure S1, which confirms that recalibration would be required before any external deployment.

Fifth, our findings rest entirely on internal validation; no external validation was performed in an independent cohort, and the discriminative estimates reported should be interpreted in that light. We addressed this limitation indirectly by triangulating three independent internal validation methods whose convergence reduces the probability that the observed performance reflects model overfitting; nonetheless, internal validation does not substitute for external validation, and we have made this position explicit throughout the manuscript.

Sixth, sensitivity and specificity values were derived from the same dataset in which the Youden-based cutoffs were identified and may therefore reflect optimism bias. The magnitude of this bias is expected to be modest given the very low AUC optimism observed in bootstrap analyses (<0.01); nonetheless, prospective external validation in independent populations will be required to obtain unbiased estimates of cutoff-based diagnostic performance.

Seventh, lifestyle factors such as habitual dietary intake, physical activity level, pre-pregnancy weight trajectory, and gestational weight gain were not systematically captured in this cohort, and therefore could not be modeled as covariates or potential mediators of the observed associations. While anthropometric indices integrate the cumulative effect of these factors on body composition at the point of measurement, the lack of explicit lifestyle data limits our ability to disentangle the relative contributions of behavioral versus constitutional determinants of GDM risk. Future prospective studies should incorporate validated dietary and activity assessment instruments to clarify these pathways. Relatedly, first-trimester biochemical markers of glucose metabolism—most notably glycated hemoglobin (HbA1c)—were not incorporated into the present analysis, and their inclusion alongside anthropometric indices in future studies may further refine early risk stratification, particularly as elevated HbA1c can occur even in women with a normal or near-normal BMI.

Eighth, as an observational design, our study evaluates whether first-trimester composite anthropometric indices predict subsequent GDM but cannot establish whether acting on this prediction—through earlier monitoring, intensified counseling, or other interventions—actually improves maternal or perinatal outcomes. This question lies outside the scope of any single-center prospective cohort study and properly belongs to a subsequent interventional or pragmatic trial.

Future Directions: Building on the limitations above, three lines of future investigation appear particularly important. First, prospective multicenter external validation in independent populations is essential to determine whether the discriminative performance and proposed cutoffs derived in this cohort transport to populations with different ethnic, anthropometric, and healthcare-system characteristics. Second, formal calibration analyses and population-specific recalibration of cutoff thresholds will be required before any clinical deployment, given the limited transportability of cutoffs demonstrated here in our direct comparison with [14]. Third, beyond demonstrating predictive performance, future work should determine whether incorporating first-trimester composite anthropometric indices into clinical decision pathways improves maternal and neonatal outcomes—through pragmatic trials of risk-stratified intensified surveillance or through implementation studies embedded in routine antenatal care. Such evidence would be required before composite anthropometric indices could be formally considered for integration into screening guidelines.

In summary, this prospective cohort study provides initial, hypothesis-generating evidence that first-trimester composite anthropometric indices—particularly BRI and ABSI—may help identify, with strong discrimination and meaningful incremental value over BMI, women at elevated risk of subsequent GDM within a specific clinical niche that current screening guidelines do not directly address: overweight pregnant women without established major risk factors. The within-cohort discriminative performance was consistent across multiple validation procedures, the reclassification gains over BMI were substantial, and decision curve analysis indicated net clinical benefit across a clinically relevant threshold range. At the same time, the findings rest on internal validation alone and were generated in a single-center Turkish cohort; the absolute discriminative estimates, the proposed cutoff values, and the implied clinical decision pathways must be tested in external populations before any change in practice could be contemplated. Conceptualized as a structured, hypothesis-generating evaluation of a specific guideline gray zone, our study offers a basis on which larger multicenter validation efforts and, in due course, interventional studies can be designed to determine whether composite anthropometric indices can be translated into measurable improvements in maternal and perinatal outcomes for this clinically distinctive subgroup.

## 5. Conclusions

In this prospective single-center cohort study of 186 overweight pregnant women without established major risk factors for gestational diabetes mellitus, first-trimester Body Roundness Index and A Body Shape Index demonstrated strong discrimination for subsequent IADPSG-defined GDM, substantially outperforming body mass index alone (AUC 0.905 and 0.889 versus 0.705; DeLong *p* < 0.001 for both pairwise comparisons). Reclassification analyses and decision curve analysis supported meaningful incremental value over a BMI-only approach, and three complementary internal validation procedures converged on highly similar estimates with optimism below 0.01. These findings provide initial, hypothesis-generating evidence that composite anthropometric indices may serve as supplementary first-trimester risk-stratification tools in a clinical gray zone—overweight pregnant women without conventional GDM risk factors—where current screening guidelines provide no explicit directive for early risk assessment. The observed effect sizes in this cohort (a BRI standardized odds ratio of 9.52 and an integrated discrimination improvement of 45.6% over BMI) are substantial; while these within-cohort estimates require cautious interpretation given the limited number of events and are expected to attenuate under external validation, their magnitude provides a reasonable quantitative rationale for pursuing larger, multicenter studies. The discriminative estimates, the proposed BRI cutoff of 4.45, and the implied clinical pathways derived from this cohort require prospective external validation in independent multicenter populations before any incorporation into clinical practice can be considered. Within the existing screening architecture, composite anthropometric indices should be regarded as potential adjuncts to—not replacements for—universal second-trimester OGTT screening.

## Figures and Tables

**Table 1:** Baseline demographic and anthropometric characteristics of the study cohort (*n* = 186).

Characteristic	Value
***Demographic***	
Age (years), median (IQR)	27.0 (25.0–32.0)
Age range (years)	18–41
Gestational age (weeks), median (IQR)	12.43 (12.00–13.00)
Gravida, median (IQR)	2 (1–3)
Parity, median (IQR)	1 (0–1)
Nulliparous, *n* (%)	78 (41.9)
Multiparous, *n* (%)	108 (58.1)
***Anthropometric***	
Height (cm), mean ± SD ^a^	162.41 ± 6.48
Weight (kg), median (IQR)	71.0 (67.0–77.0)
Body mass index (kg/m^2^), median (IQR)	26.86 (25.46–29.12)
BMI range (kg/m^2^)	25.00–29.78
Waist circumference (cm), median (IQR)	85.0 (80.0–89.0)
Body Roundness Index (BRI), median (IQR)	3.77 (3.24–4.34)
BRI range	1.83–7.08
A Body Shape Index (ABSI), median (IQR)	0.0737 (0.0707–0.0768)
ABSI range	0.0620–0.0910
***Outcome***	
GDM-positive, *n* (%)	34 (18.28)
GDM-negative, *n* (%)	152 (81.72)

Continuous variables were assessed for normality using the Shapiro–Wilk test. ^a^ Height was the only continuous variable with normal distribution (*p* = 0.061) and is therefore presented as mean ± standard deviation; all other continuous variables were non-normal (*p* < 0.05) and are presented as median (interquartile range). GDM = gestational diabetes mellitus; BMI = body mass index; BRI = Body Roundness Index; ABSI = A Body Shape Index; IQR = interquartile range; SD = standard deviation.

**Table 2:** Comparison of baseline and anthropometric characteristics between GDM-positive (*n* = 34) and GDM-negative (*n* = 152) groups.

Characteristic	GDM-Positive (*n* = 34)	GDM-Negative (*n* = 152)	*p*-Value	Effect Size
***Demographic***				
Age (years) ^a^	28.5 (25.0–33.0)	27.0 (25.0–32.0)	0.255	*r* = 0.08
Gestational age (weeks) ^a^	12.36 (11.89–13.00)	12.43 (12.00–13.00)	0.792	*r* = 0.02
Gravida ^a^	2 (1–3)	2 (1–3)	0.444	*r* = 0.06
Parity ^a^	1 (0–1.75)	1 (0–1)	0.236	*r* = 0.09
Nulliparous, *n* (%) ^b^	12 (35.3)	66 (43.4)	0.499	—
***Anthropometric***				
Height (cm) ^a^	160.0 (157.0–163.0)	162.0 (158.0–168.0)	0.113	*r* = 0.12
Weight (kg) ^a^	74.0 (68.5–78.0)	71.0 (66.0–76.0)	0.228	*r* = 0.09
BMI (kg/m^2^) ^a^	28.91 (26.73–29.65)	26.47 (25.30–28.81)	**<0.001**	***r* = 0.27**
Waist circumference (cm) ^a^	95.0 (89.0–99.75)	83.5 (79.75–87.0)	**<0.001**	***r* = 0.49**
Body Roundness Index ^c^	5.17 ± 0.98	3.60 ± 0.72	**<0.001**	***d* = 2.04**
A Body Shape Index ^c^	0.080 ± 0.005	0.072 ± 0.004	**<0.001**	***d* = 1.83**

Values are median (interquartile range) or *n* (%) unless otherwise indicated. ^a^ Compared using the Mann–Whitney U test due to non-normal distribution within at least one group; effect size reported as *r* = Z/√N. ^b^ Compared using the chi-square test. ^c^ Normally distributed within both groups (Shapiro–Wilk *p* > 0.05); presented as mean ± standard deviation and compared using the independent samples t-test; effect size reported as Cohen’s *d*. GDM = gestational diabetes mellitus; BMI = body mass index. Bold *p*-values indicate statistical significance (*p* < 0.05).

**Table 3:** Diagnostic performance of first-trimester anthropometric indices for prediction of subsequent IADPSG-defined gestational diabetes mellitus (*n* = 186; 34 GDM-positive).

Predictor	AUC (95% CI) ^a^	Cutoff ^b^	Sensitivity	Specificity	PPV	NPV	LR+	LR−
Body mass index (kg/m^2^)	0.705 (0.601–0.810)	28.04	67.6%	67.8%	31.9%	90.4%	2.10	0.48
Waist circumference (cm)	0.865 (0.784–0.946)	88.00	82.4%	79.6%	47.5%	95.3%	4.04	0.22
**Body Roundness Index**	**0.905 (0.835–0.974)**	**4.45**	**85.3%**	**90.8%**	**67.4%**	**96.5%**	**9.26**	**0.16**
A Body Shape Index	0.889 (0.815–0.963)	0.0772	73.5%	90.1%	62.5%	93.8%	7.45	0.29
**Pairwise Comparison (DeLong’s Test)**			**ΔAUC**			***p*** **-Value**		
BRI vs. BMI			**+0.199**			**<0.001**		
ABSI vs. BMI			**+0.184**			**<0.001**		
Waist circumference vs. BMI			**+0.159**			**<0.001**		
BRI vs. ABSI			+0.016			0.462		
BRI vs. waist circumference			+0.040			0.071		
ABSI vs. waist circumference			+0.024			0.124		

^a^ Area under the ROC curve (AUC) with 95% confidence interval calculated using the Hanley and McNeil method. ^b^ Optimal cutoff determined by the Youden index (J = sensitivity + specificity − 1). AUCs were compared between predictors using DeLong’s test for paired ROC curves. ΔAUC values were computed from unrounded AUCs and may therefore differ in the second decimal from differences computed using the rounded values displayed in the table. AUC = area under the receiver operating characteristic curve; PPV = positive predictive value; NPV = negative predictive value; LR+ = positive likelihood ratio; LR− = negative likelihood ratio. Bold values indicate the best-performing index.

**Table 4:** Univariate and multivariate logistic regression analyses of demographic and anthropometric predictors of subsequent GDM (*n* = 186; 34 GDM-positive).

Predictor	Standardized OR (per 1 SD) ^a^		95% CI	*p*-Value
***Univariate analyses***				
Age	1.29		0.89–1.87	0.177
BMI	2.08		1.38–3.14	<0.001
Waist circumference	5.42		3.09–9.51	<0.001
A Body Shape Index	7.91		3.92–15.97	<0.001
**Body Roundness Index**	**9.52**		**4.63–19.56**	**<0.001**
***Multivariate Model A ^b^***				
Age (per 1 SD)	1.04		0.64–1.69	0.885
**Body Roundness Index (per 1 SD)**	**9.44**		**4.56–19.54**	**<0.001**
***Multivariate Model B ^c^***				
Age (per 1 SD)	1.02		0.61–1.68	0.863
BMI (per 1 SD)	1.81		1.07–3.05	0.028
**A Body Shape Index (per 1 SD)**	**7.13**		**3.49–14.54**	**<0.001**
**Model Performance**	**AUC**	**AIC**	**Pseudo R^2^**	**VIF Range ^d^**
Model A (Age + BRI)	0.9044	104.76	0.4418	1.02–1.02
Model B (Age + BMI + ABSI)	0.8996	112.11	0.4116	1.04–1.11

^a^ Standardized odds ratios per one standard deviation increment, calculated from logistic regression on standardized variables to enable direct comparison of effect sizes across predictors with different measurement scales. ^b^ Model A combines age and BRI. BMI and BRI were not entered together in this primary model because their joint inclusion produced unstable, sign-reversed coefficients consistent with statistical suppression, reflecting their substantial empirical correlation in this cohort; the incremental combination of BMI and a shape-based index was instead assessed in the reclassification and decision-curve analyses. ^c^ Model B combines age, BMI, and ABSI; ABSI is, by mathematical construction, designed to be largely independent of BMI. ^d^ Variance inflation factors (VIF) were calculated from properly specified models with intercept terms on standardized variables. All VIFs were below 2.0, indicating no concerns regarding multicollinearity in either model. AUC = area under the receiver operating characteristic curve; AIC = Akaike Information Criterion; OR = odds ratio; CI = confidence interval; SD = standard deviation; VIF = variance inflation factor. Bold values indicate statistical significance (*p* < 0.05).

**Table 5:** Internal validation of model performance using three independent procedures (*n* = 186; 34 GDM-positive).

Predictor/Model	Apparent AUC ^a^	Bootstrap-Corrected AUC ^b^	Optimism	5-Fold CV AUC ^c^	LOOCV AUC ^d^	Brier Score ^e^
***Univariate predictors***						
Body Roundness Index	0.9047	0.9041	+0.0006	0.8993 (±0.0438)	0.8973	0.072
A Body Shape Index	0.8891	0.8908	−0.0017	0.8913 (±0.0460)	0.8796	0.086
***Multivariate models***						
Model A (Age + BRI)	0.9044	0.9022	+0.0022	0.8961 (±0.0469)	0.8953	0.071
Model B (Age + BMI + ABSI)	0.8996	0.8929	+0.0066	0.8812 (±0.0452)	0.8849	0.079

^a^ Apparent AUC: area under the ROC curve computed on the same data used to fit the model. ^b^ Bootstrap-corrected AUC: derived from 1000 bootstrap resamples following the optimism-correction procedure; optimism estimates the expected over-fit. ^c^ Stratified 5-fold cross-validation: data partitioned with preservation of the GDM event rate within each fold; values shown as mean (±SD across folds). ^d^ Leave-one-out cross-validation: each participant in turn held out as the test sample while the model is re-fit on the remaining *n* − 1 participants. ^e^ Brier score: mean squared difference between predicted probabilities and observed outcomes; lower values indicate better calibration. Optimism values below 0.01 across all models, together with close convergence of the three independent validation procedures, indicate that the observed discriminative performance is unlikely to reflect substantial overfitting within this cohort. AUC = area under the receiver operating characteristic curve; CV = cross-validation; LOOCV = leave-one-out cross-validation; SD = standard deviation.

## Data Availability

The data presented in this study are available on request from the corresponding author. The data are not publicly available due to privacy and ethical restrictions imposed by the institutional review board.

## Supplementary Materials

The following supporting information can be downloaded at https://www.mdpi.com/article/10.3390/diagnostics16152384/s1, Table S1: STARD 2015 checklist, Figure S1: calibration plot for the primary model.
