# Supplementary material for: Body Roundness Index and A Body Shape Index as First-Trimester Risk-Stratification Markers for Gestational Diabetes in Overweight Pregnant Women Without Established Major Risk Factors: A Prospective Single-Center Cohort Study
Source: Diagnostics (Basel). 2026 Jul 29;16(15):2384. doi: 10.3390/diagnostics16152384 (PMC13466089; doi:10.3390/diagnostics16152384)
Supplement: Supplementary file 1 [file diagnostics-16-02384-s001.zip › Supplementary Table S1. STARD.pdf]

Supplementary Table S1. STARD 2015 Checklist

Standards for Reporting of Diagnostic Accuracy Studies (STARD) 2015 checklist for the manuscript "Body Roundness Index and A Body Shape Index as First-Trimester Risk-Stratification Markers for Gestational Diabetes in Overweight Pregnant Women Without Established Major Risk Factors: A Prospective Single-Center Cohort Study."

Section	Item #	STARD 2015 Checklist Item	Reported on (section / line)
**Title or Abstract**	1	Identification as a study of diagnostic accuracy using at least one measure of accuracy (such as sensitivity, specificity, predictive values, or AUC).	Title (page 1); Abstract (Results: AUC, sensitivity, specificity)
	2	Structured summary of study design, methods, results, and conclusions.	Abstract (Background/Objectives, Methods, Results, Conclusions)
**Introduction**	3	Scientific and clinical background, including the intended use and clinical role of the index test.	Introduction, ¶1–¶8 (clinical gray zone rationale; intended use as supplementary first-trimester risk-stratification tool)
	4	Study objectives and hypotheses.	Introduction, final paragraph (a priori hypothesis; primary and secondary objectives)
**Methods**	5	Whether data collection was planned before the index test and reference standard were performed (prospective study) or after (retrospective study).	Methods 2.1 (prospective, observational cohort; data collection planned a priori)
	6	Eligibility criteria.	Methods 2.2 (Inclusion criteria; Exclusion criteria)
	7	On what basis potentially eligible participants were identified (such as symptoms, results from previous tests, inclusion in a registry).	Methods 2.2 (consecutive women presenting to any of eleven parallel general obstetric outpatient clinics (4,284 women screened) and meeting predefined eligibility criteria)
	8	Where and when potentially eligible participants were identified (setting, location, and dates).	Methods 2.2 (Ankara Etlik City Hospital; eleven general obstetric outpatient clinics; 9–19 February 2026)
	9	Whether participants formed a consecutive, random, or convenience series.	Methods 2.2 (consecutive enrollment on every working day during the enrollment period)
	10a	Index test, in sufficient detail to allow replication.	Methods 2.3 (standardized anthropometric measurements; BRI and ABSI calculation formulas)

Section	Item #	STARD 2015 Checklist Item	Reported on (section / line)
	10b	Reference standard, in sufficient detail to allow replication.	Methods 2.4 (75-g OGTT protocol; laboratory method) and 2.5 (IADPSG diagnostic criteria)
	11	Rationale for choosing the reference standard (if alternatives exist).	Methods 2.1 and 2.5 (IADPSG one-step 75-g OGTT as the internationally adopted reference standard)
	12a	Definition of and rationale for test positivity cut-offs or result categories of the index test, distinguishing prespecified from exploratory.	Methods 2.6 (ROC Analysis; optimal cut-offs derived by the Youden index — data-derived and exploratory, reported as a cohort-specific analytic anchor rather than a transferable clinical threshold)
	12b	Definition of and rationale for test positivity cut-offs or result categories of the reference standard, distinguishing prespecified from exploratory.	Methods 2.5 (prespecified IADPSG glucose thresholds: FPG ≥92, 1-h ≥180, 2-h ≥153 mg/dL)
	13a	Whether clinical information and reference standard results were available to the performers or readers of the index test.	Methods 2.5 (nurses performing anthropometry were blinded to subsequent OGTT outcomes)
	13b	Whether clinical information and index test results were available to the assessors of the reference standard.	Methods 2.5 (attending obstetricians reviewing OGTT were not involved in anthropometric assessment)
	14	Methods for estimating or comparing measures of diagnostic accuracy.	Methods 2.6 (ROC Analysis; Hanley–McNeil 95% CI; DeLong's test; logistic regression; reclassification metrics)
	15	How indeterminate index test or reference standard results were handled.	Methods 2.2 (no indeterminate results; 12 participants without outcome data excluded with reasons specified)
	16	How missing data on the index test and reference standard were handled.	Methods 2.2 and Results 3.1 (complete-case analysis; 12 participants with missing outcome excluded; reasons reported)
	17	Any analyses of variability in diagnostic accuracy, distinguishing prespecified from exploratory.	Methods 2.6 (Subgroup Analyses by predefined BMI subgroups, prespecified); Results 3.8
	18	Intended sample size and how it was determined.	Methods 2.6 (Sample Size Determination; a priori G*Power analysis; n = 186)

Section	Item #	STARD 2015 Checklist Item	Reported on (section / line)
**Results**	19	Flow of participants, using a diagram.	Results 3.1; Figure 1 (STARD flow diagram)
	20	Baseline demographic and clinical characteristics of participants.	Results 3.2; Table 1
	21a	Distribution of severity of disease in those with the target condition.	Results 3.3 (Table 2, GDM-positive group) and 3.8 (GDM distribution across BMI subgroups)
	21b	Distribution of alternative diagnoses in those without the target condition.	Not applicable (binary outcome: GDM-positive vs GDM-negative by IADPSG criteria); GDM-negative group characteristics in Table 2
	22	Time interval and any clinical interventions between index test and reference standard.	Methods 2.3/2.4 (index test at 11+0–13+6 weeks; reference standard at 24+0–28+0 weeks; no interventions — observational design)
	23	Cross-tabulation of the index test results (or their distribution) by the results of the reference standard.	Results 3.4; Table 3 (sensitivity, specificity, PPV, NPV, LR+, LR− by GDM status)
	24	Estimates of diagnostic accuracy and their precision (such as 95% confidence intervals).	Results 3.4; Table 3 (AUCs with 95% CI); Table 4 (ORs with 95% CI)
	25	Any adverse events from performing the index test or the reference standard.	Methods 2.1 (non-invasive anthropometry; routine 75-g OGTT imposed no additional procedure or risk; no adverse events)
**Discussion**	26	Study limitations, including sources of potential bias, statistical uncertainty, and generalizability.	Discussion, Limitations (eight explicitly enumerated limitations)
	27	Implications for practice, including the intended use and clinical role of the index test.	Discussion (practical implications; augmentation-not-replacement framing); Conclusions
**Other Information**	28	Registration number and name of registry.	Not registered as a clinical trial (observational study); ethics approval AEŞH-EK-2026-035 (4 February 2026)
	29	Where the full study protocol can be accessed.	Data Availability Statement (study protocol and data available from the corresponding author on reasonable request)

Section	Item #	STARD 2015 Checklist Item	Reported on (section / line)
	30	Sources of funding and other support; role of funders.	Funding statement (this research received no external funding)

AUC = area under the receiver operating characteristic curve; BMI = body mass index; BRI = Body Roundness Index; ABSI = A Body Shape Index; OGTT = oral glucose tolerance test; IADPSG = International Association of Diabetes and Pregnancy Study Groups; FPG = fasting plasma glucose; PPV = positive predictive value; NPV = negative predictive value; LR+ = positive likelihood ratio; LR− = negative likelihood ratio; CI = confidence interval; OR = odds ratio.

Reference: Bossuyt PM, Reitsma JB, Bruns DE, et al. STARD 2015: An Updated List of Essential Items for Reporting Diagnostic Accuracy Studies. Radiology 2015;277:826–832.
